# Dynamical and Thermodynamical Influences of the Maritime Continent on ENSO Evolution

**DOI:** 10.1038/s41598-018-33436-5

**Published:** 2018-10-18

**Authors:** Tuantuan Zhang, Bohua Huang, Song Yang, Junwen Chen, Xingwen Jiang

**Affiliations:** 1 School of Atmospheric Sciences, Sun Yat-sen University, Guangzhou, China; 2 Department of Atmospheric, Oceanic, and Earth Sciences and Center for Ocean-Land-Atmosphere Studies, George Mason University, Fairfax, Virginia USA; 3 Guangdong Province Key Laboratory for Climate Change and Natural Disaster Studies, Sun Yat-sen University, Guangzhou, China; 4 Institute of Earth Climate and Environment System, Sun Yat-sen University, Guangzhou, China; 5 Institute of Plateau Meteorology, China Meteorological Administration, Chengdu, China

## Abstract

El Niño-Southern Oscillation (ENSO) exerts tremendous influences on the global climate. Through dynamic lifting and thermal forcing, the Maritime Continent (MC) plays an important role in affecting global atmospheric circulation. In spite of the extensive studies on ENSO mechanisms, the influence of MC on the characteristics of ENSO life cycle remains unclear. Our coupled model experiments reveal that the absence of the MC land contributes to a strong ENSO asymmetry and a weakened nonlinear atmospheric response to the combined seasonal and interannual SST variations (i.e. the combination mode) that prolongs the warm events, resulting in a reduction of ENSO frequency. On the other hand, our experiments suggest that the global climate model applied (NCAR CESM) overestimates the MC topographic uplifting effect on ENSO simulation. Overall, this study provides a new physical insight into the nature of the MC influence on ENSO evolution.

## Introduction

El Niño-Southern Oscillation (ENSO) is a major driver for global interannual climate variability^1,2^. It exerts worldwide impacts on human life and economic growth through modulating crop yields^3^, drought and flood hazards^4,5^, heat waves and cold surge^6^, tropical cyclones^7^, and ice melting in polar regions^8,9^. Understanding, simulation, and prediction of ENSO have attracted tremendous interests around the world over past few decades. Among many challenges, the future change of ENSO in a warming climate is thought to be at the heart of global climate change research^10,11^.

The warm (El Niño) or cold (La Niña) episodes of ENSO occur every 2–7 years^12^. These events typically follow a similar life cycle, initiating in boreal spring, developing during boreal summer and fall, peaking in boreal winter, and decaying from the subsequent spring to early summer^13,14^. Physically, a fast ENSO growth is associated with a coupled positive feedback among the zonal sea surface temperature (SST) gradient, surface zonal wind, and the zonal slope of thermocline depth over the equatorial Pacific, characterized by a zonal shift of the Walker circulation^15^. On the other hand, delayed negative feedback mechanisms, such as the delayed action oscillator^16^ and the meridional discharge-recharge process^17,18^, generate phase transitions in a sustained ENSO cycle. Other physical processes further account for ENSO’s phase locking with the annual cycle^19^ as well as the quick termination of an ENSO event after its peaking phase^20,21^.

Oceanographically, the Maritime Continent (MC) and its associated island chains form the western boundary of the Pacific, which, among other things, allows the reflection of the incoming oceanic Rossby waves into eastward propagating equatorial Kelvin waves, a crucial process of the ENSO delayed oscillator^16^. The Indonesian Throughflow also transports warm and fresh Pacific water into the Indian Ocean^22^. Meteorologically, atmospheric deep convection over the western Pacific warm pool, which fuels the ascending branches of both the Walker cell and the meridional cell, has its core anchored around the MC^23^. During the ENSO warm episode, convection is depressed over the MC and enhanced near the dateline, accompanying an eastward shift of the Walker circulation, while the features are opposite during the cold episode^24,25^. Previous studies have suggested that the interannual climate variation over the MC is significantly modulated by ENSO^26–29^. However, as a major tropical heat source located between the Indian Ocean and the western Pacific, the MC plays a critical role in bridging the global atmospheric circulations, including forming the ascending branches of the meridional cell and Walker cell^23^, suggesting that the MC may be not only a respondent but also a driver. Despite that much effort has been devoted to this fascinating place over the recent decades^23,30,31^, how the MC influences the characteristics of the ENSO cycle remains far from understanding.

Consisting of multiple islands in Southeast Asia with complex topographies, the MC affects the atmospheric circulation through its topographic dynamic lifting and land surface thermal properties that are distinctive to those over the surrounding oceans. To examine the MC influences as both a dynamical and a thermal forcing on the characteristics of ENSO life cycle, we perform three coupled numerical experiments from 1979 to 2005 using the Community Earth System Model (CESM, see Methods for details). The first experiment uses the U.S. Geological Survey (USGS) 30-second elevation over the globe (referred to as CTL, Supplementary Fig. 1a), while the elevation is reduced to zero over the MC in the second experiment, to highlight the topographic effect which is dominated by dynamical lifting (referred to as NOTOPO, Supplementary Fig. 1b). Finally, to show the combined effect of MC topography and thermal forcing, the land surface over the MC is replaced by a layer of seawater of 10-meter depth in the third experiment (referred to as NOLAND). The 10-meter depth is only the thickness of the OGCM first layer and does not generate any meaningful transport through it. Therefore, it does not change the large-scale ocean circulation including the differences of sea surface temperature and salinity between the Western Pacific and Indian Ocean. However, the change of the surface property from landmass to seawater is sufficient for atmosphere-ocean interaction to take effect. We focus on the period from 1983 to the end of the simulations.

## Results

### Changes in frequency band and life cycle of ENSO

**Figure 1:**
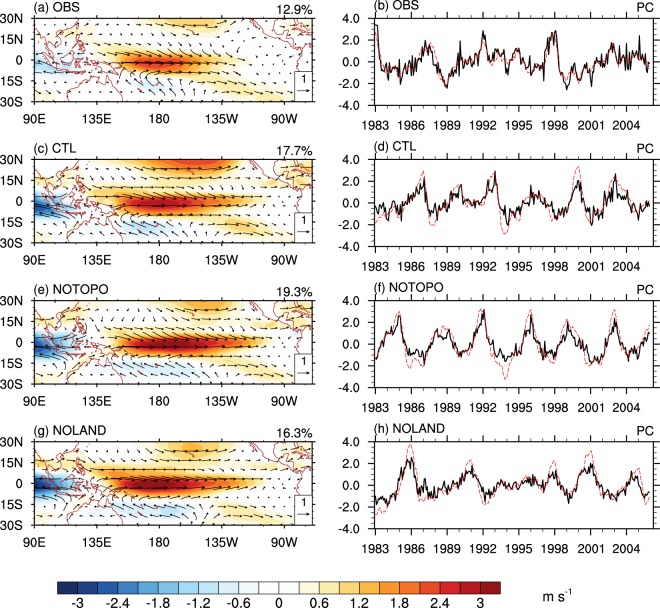
First combined EOF modes of 925-hPa wind, representing the ENSO mode. (left) Spatial pattern and (right) corresponding principal component (PC) for (**a**,**b**) NCEP CFSR, (**c**,**d**) CTL experiment, (**e**,**f**) NOTOPO experiment, and (**g**,**h**) NOLAND experiment, respectively. Shadings in the left panels represent the pattern of zonal wind. In the right panels, solid black lines denotes the corresponding PC1s, and dashed red lines denotes Niño-3.4 indices.

The first combined empirical orthogonal function (CEOF) mode of the observed 925-hPa wind within the tropical Indo-Pacific domain accounts for 12.9% of the total variance. This dominant pattern of surface wind variability is characterized by opposite zonal wind anomalies over the equatorial western-central Pacific and the equatorial Indian Ocean (i.e. Walker circulation anomalies, Fig. 1a). A pair of anomalous cyclonic/anticyclone circulation is situated over each side of the anomalous wind over the equatorial Pacific (Fig. 1a). This spatial pattern apparently represents the atmospheric anomalies associated with ENSO. Its corresponding principal component (PC1 hereafter) is highly correlated with Niño-3.4 index (SST anomalies over 5°S–5°N, 120°W–170°W), with a correlation coefficient of 0.84.

The major features of the observed CEOF mode are generally captured by the CESM (Fig. 1a,c), giving confidence in exploring the role of MC in ENSO life cycle through additional model experiments. The NOTOPO (Fig. 1e) and NOLAND (Fig. 1g) experiments reveal similar spatial patterns to those of the CTL experiment (Fig. 1c). A low-frequency fluctuation about 3–4 years is shown in the PC1 of the CTL experiment (Fig. 1d), in the time scale of the observed ENSO interannual variability^12,32^. The correlation coefficient between Niño-3.4 index and the PC1 in the CTL experiment is 0.83, suggesting that the PC1 well captures ENSO activity in the CTL experiment. The model ENSO cycle tends to be quite regular (Fig. 1d). No significant change in ENSO interannual variability is found when the MC topography is removed (Fig. 1f). However, the frequency and strength of ENSO are reduced when the thermal property of the MC surface is further changed from land to water (Fig. 1h). These features of NOLAND are confirmed by the power spectrum of the PC1s (blue curve, supplementary Fig. 2a) in comparison to those from the other two experiments (red and orange curves, Supplementary Fig. 2a). The power spectrum of the Niño-3.4 index reinforces the result in Supplementary Figs 2 and 3. Visually, El Niño events decay rapidly after their peaks in both CTL (Fig. 1d) and NOTOPO (Fig. 1f) experiments, which are similar to the observed major events in 1983, 1992, and 1998 (Fig. 1b). However, the decaying process seems slower in the NOLAND El Niño events (Fig. 1h), which may contribute to its lower frequency of ENSO cycle.

**Figure 2:**
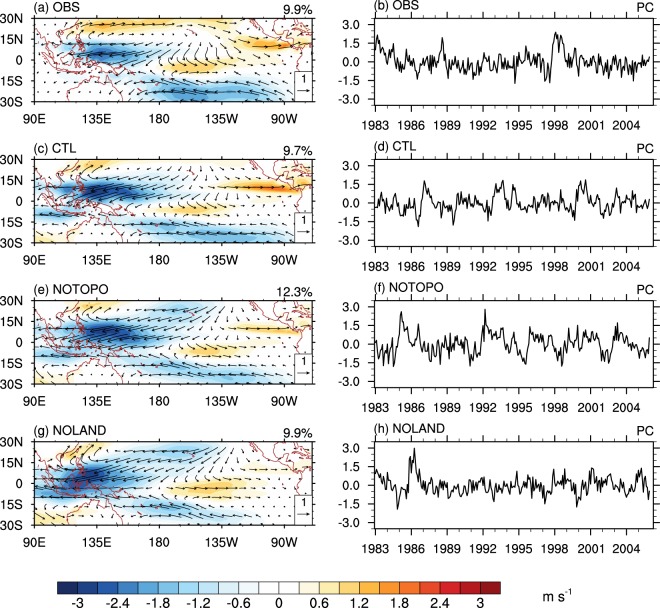
Second combined EOF modes of 925-hPa wind, representing the combined mode. Same as Fig. 1, but for the second leading combination EOF modes.

The second CEOF mode from observations, which explains 9.9% of total variance, is characterized by an anomalous anticyclonic circulation over the western North Pacific (WNP) and anomalous westerlies to the south of the equator over the central Pacific (Fig. 2a). These anomalies are particularly strong in the maturing season of major El Niño events in the boreal spring of 1983 and 1998 when the positive values of the corresponding time series (PC2 hereafter) are large (Fig. 2b). This pattern of low-level wind anomaly features the combination mode (C-mode) generated by the nonlinear interaction between the warm pool annual cycle and the ENSO interannual variability^20,33–35^. The PC2 spectrum shows quasi-annual frequencies with two significant peaks around 15 months and 9 months (Supplementary Fig. 2b; Stuecker *et al*.)^20^ as the combination tones in the C-mode. The two significant peaks are captured by both the CTL and NOTOPO experiments, although there are some overestimations (Fig. 2). However, the PC2 of the NOLAND experiment (Fig. 2h) does not show distinctive maxima as in the other two runs. Correspondingly, its power spectrum only shows a small peak of the combination mode frequency around 9 months (blue curve, Supplementary Fig. 2b). Since the C-mode is effective in generating the rapid termination of strong El Niño events, this result further confirms that the combined effect of MC topography and thermal forcing plays an important role in affecting El Niño decaying process and modulating ENSO frequency.

**Figure 3:**
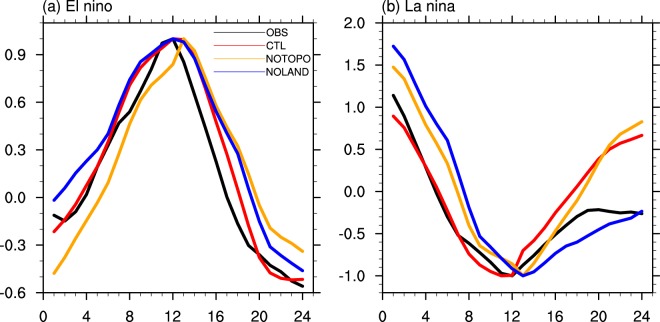
Composite of El Niño and La Niña events. Composite patterns of 3-month running mean Niño-3.4 SST anomalies in (**a**) El Niño and (**b**) La Niña events in observation (black line), CTL experiment (red line), NOTOPO experiment (orange line), and NOLAND experiment (blue line). El Niño (La Niña) events are defined as 3 month running mean of Niño-3.4 SST anomaly exceeding 0.5 (−0.5) for 5 consecutive overlapping seasons. The x-coordinate indicates corresponding months from ENSO Year 0 to Year 1. The curves are normalized by dividing each with its peak value.

To further substantiate the changes in ENSO life cycle, we compute the composite values for the El Niño and La Niña events from Year 0 to Year 1 (Fig. 3). The El Niño (La Niña) events are selected with 3-month running mean Niño-3.4 index exceeding 0.5 (−0.5) for 5 consecutive overlapping seasons. The curves are normalized by dividing each with its peak value (i.e. during the peak of El Nino or La Nina). The El Niño composite values for both CTL and NOLAND experiments peak in December of Year0 while the latter clearly shows a slower decay (Fig. 3a). On the other hand, the composite El Niño value for NOTOPO experiment peaks later. Considering that the NOTOPO event also initiates somewhat later than in CTL and NOLAND, its phase-locking character seems to be different from the other two. A similar shift in peaking time also occurs in the La Niña composite for NOTOPO experiment (Fig. 3b). It is inconclusive whether the ENSO decay in the NOTOPO experiment is clearly faster than that in the CTL experiment although its C-mode is stronger. A new finding from the composite analysis is that the decays of the La Niña events are much slower in the NOLAND experiment compared to those in both CTL and NOTOPO experiments (Fig. 3b). Interestingly, the NOLAND La Niña decaying is much closer to the observations than the other two are. The strong ENSO asymmetry in the NOLAND experiment apparently contributes to the slower decay of the ENSO events, especially the cold events, and hence a lower frequency of ENSO cycle, in addition to the C-mode mechanism.

### Physical influences of MC on ENSO decaying process

**Figure 4:**
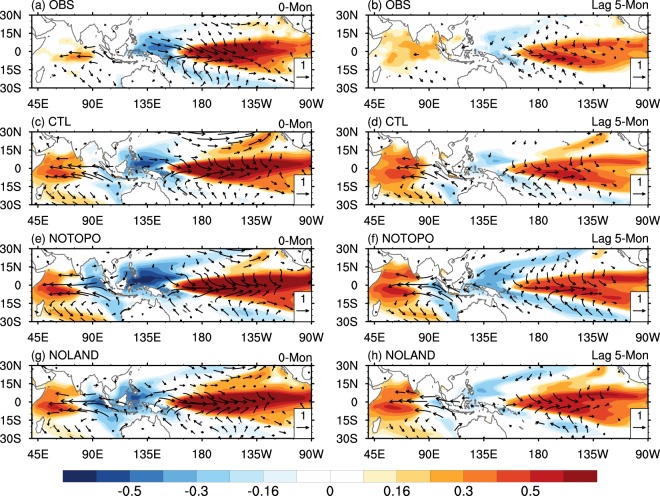
Ocean-atmosphere anomalies related to ENSO life cycle. Correlations of SST and 10 m wind with the first PCs for (left) lag 0-month and (right) lag 5-month from (**a**,**b**) NCEP CFSR, (**c**,**d**) CTL experiment, (**e**,**f**) NOTOPO experiment, and (**g**,**h**) NOLAND experiment, respectively. Significant values exceeding the 90% confidence level are shown.

ENSO growth is generated by the positive ocean-atmosphere feedback in the equatorial Pacific (Bjerknes feedback), leading to persistent development of the equatorial anomalies and finally reaching its peak phase^15^. As shown in Supplementary Fig. 4, warm SST and surface wind anomalies over the tropical eastern-central Pacific cold tongue are largest five months before the ENSO peaks in the NOTOPO experiment but smallest in the NOLAND experiment, implying a faster growth and thus larger peak values in the former. Consistently, weakest (strongest) Bjerknes feedback occurs in the NOLAND (NOTOPO) experiment, which leads to the slowest (fastest) ENSO development (figure not shown). At the peak phase of El Niño, SST increases over the tropical eastern-central Pacific cold tongue and decreases over the western Pacific warm pool, with a reduced east-west SST gradient and weakened equatorial easterly wind, resulting in a slacken Walker circulation (Fig. 4a). In that stage, similar features are shown among the three experiments, except that the cold SST anomalies over the WNP region are more prominent in the NOTOPO experiment, demonstrating a stronger regional air-sea thermodynamic feedback in the western North Pacific, which strengthens the anomalous WNP anticyclonic circulation^36^ (WNP_AC). The positive (negative) SST anomalies over the tropical eastern-central Pacific cold tongue (WNP warm pool) decay during the subsequent months, while warm SST anomalies develop over the Indian Ocean (Fig. 4b). Correspondingly, the WNP-AC develops and persists into the post-ENSO summer, accompanied by a southward shift of low-level anomalous westerlies over the central pacific as part of the C-mode (Fig. 4b). In fact, the positive SST anomalies decay slower in the NOLAND experiment compared to those in the CTL and NOTOPO experiments.

**Figure 5:**
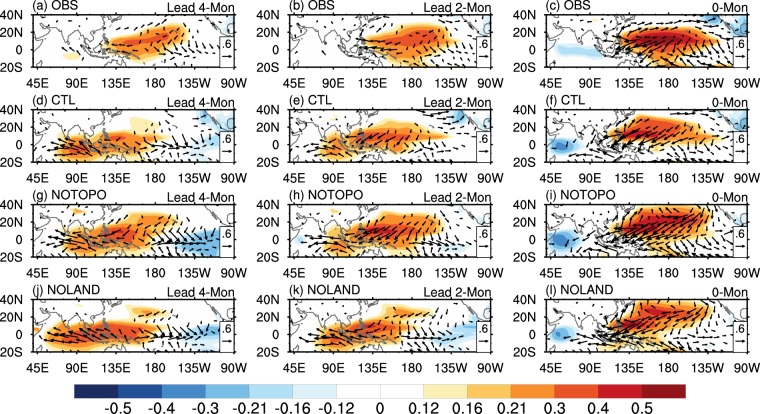
ENSO decaying process. Correlations of SLP and 10 m wind with the second PCs for (left) lead 4-month, (middle) lead 2-month, and (right) lag 0-month from (**a**–**c**) NCEP CFSR, (**d–f**) CTL experiment, (**g–i**) NOTOPO experiment, and (**j–l**) NOLAND experiment, respectively. Significant values exceeding the 90% confidence level are shown.

To further elucidate the role of MC in the ENSO decaying process, lagged correlations of sea level pressure (SLP) and 10 m wind with the PC2s are calculated (Fig. 5). In 4 months before the PC2 peak (4-month lead hereafter), the associated correlation patterns are largely asymmetric with respect to the equator, documented by the prominent positive SLP anomalies and a weak anomalous anticyclonic circulation over the WNP, with just a slight southward shift of anomalous westerlies (Fig. 5a). During the subsequent months, the anomalous WNP-AC continues to develop with a northeastward expansion, and the anomalous westerlies shift further southward to around 10°S (Fig. 5b,c). This typical C-mode dynamics modulates the equatorial heat content recharge/discharge process and generates eastward propagating upwelling Kelvin waves, allowing the thermocline to adjust toward a normal state in both western and eastern Pacific, thus favors the rapid termination of El Niño events^20,37,38^. On the other hand, the C-mode dynamics is less pronounced for La Niña as well as weak El Niño events, as in (refs^19,20^).

A delayed establishment and weaker anomalous of WNP-AC are presented in the CESM simulations compared to those observed, with a quasi-symmetric anomalous pattern at 4-month lead, and the anomalous WNP-AC establishes until 2-month lead (Fig. 5d–f). The anomalous WNP-AC establishes at 4-month lead with a larger magnitude in the NOTOPO experiment compared to that in the CTL experiment (Fig. 5d–i), demonstrating the C-mode features in the NOTOPO experiment are closer to those in observation and suggesting that the dynamical uplifting may be too strong in the coupled model. On the other hand, the establishment of the anomalous WNP-AC is further delayed (the establishment of WNP-AC occurs at later than 2-month lead) in the absence of MC dynamical forcing and thermal forcing, accompanied by a weaker southward anomalous wind shift (Fig. 5j–l). This feature signifies a weakened C-mode that prolongs the warm events and hence reduces ENSO frequency, supporting the analyses of the second CEOF mode as discussed previously.

### Possible mechanisms responsible for the change in C-mode dynamics

The decaying process of a warm event is governed by the C-mode dynamics. In addition, cold SST anomalies preceding a peak of C-mode over the western tropical Pacific favor the occurrence and amplification of the further-westward WNP-AC as a Rossby wave response, which increases SST^36,39^ (Supplementary Fig. 5a–c). The CESM simulates weaker cold SST anomalies compared to those in the observation after 4-month lead, possibly explaining a weaker and delayed establishment of the anomalous WNP-AC in the model as a weaker air-sea feedback described by (ref.^36^) (Supplementary Fig. 5d–f). Eliminating the MC topographical effect brings the features of cold SST anomalies over the western tropical Pacific closer to observation and contributes to a more realistic simulation of the anomalous WNP-AC and hence the C-mode (Supplementary Fig. 5g–i). Besides, the excessive climatological ascending motion in the CESM simulations seems to generate extra cyclonic circulation in the lower atmosphere, which weakens the anticyclonic circulation associated with the seasonal westward extension of the subtropical high (Supplementary Fig. 6b). As a result, the seasonal evolution of climate in this region may not be conducive to triggering the C-mode dynamics (Supplementary Fig. 6). Indeed, eliminating the MC topographical effect actually strengthens the subtropical high in November and leads to a more prominent WNP-AC (Supplementary Fig. 6).

Besides the cold SST anomalies over the western tropical Pacific, warm SST anomalies are observed over the MC region at 2-month lead (Supplementary Fig. 5a–c). Although these warm SST anomalies are well simulated in both CTL and NOTOPO experiments, it is absent in the NOLAND experiment until 0-month lead (Supplementary Fig. 5d–l). The establishment of the anomalous WNP-AC is also delayed in the NOLAND experiment, compared to that in the CTL experiment. This result suggests that the thermal forcing of MC as land surface plays a critical role in the establishment of the anomalous WNP-AC and hence the C-mode dynamics.

## Discussion and Concluding Remarks

Understanding the cause of ENSO seasonal synchronization is of central importance to ENSO prediction. However, we are still facing great challenges particularly in understanding the ENSO decaying process^13,14,20^. The physical mechanisms that governing the quick ENSO termination have been discussed in previous studies and they include the C-mode theory^20^ and the reversal of zonal heat transport dictated by oceanic dynamics^21^.

Results from coupled numerical model experiments show that an improved ENSO simulation in the C-mode dynamics is enhanced in the absence of the MC topographical effect, despite the unrealistic nature of the NOTOPO experiment. The overestimate ascending motion in the CTL experiment generates extra climatological cyclonic circulation in the lower atmosphere and weakens the seasonal westward extension of the subtropical high to trigger the C-mode dynamics, which is weaker and closer to that in observation in the NOTOPO experiment. Additionally, the eliminating MC topographical effect produces a better simulation of cold SST anomalies over the western tropical Pacific, which also leads to a better representation of the C-mode dynamics. This result suggests an important role of cold SST anomalies in the development of anomalous WNP-AC, consistent with finding by (ref.^36^).

On the other hand, when the effects of MC topography and thermal forcing are both excluded, the model simulates a weakened C-mode dynamics that prolongs the warm events and results in a reduction of ENSO frequency. In the NOLAND experiment, cold SST anomalies over the western tropical Pacific are well reproduced. However, the simulated MC warm SST anomalies are delayed by about 2-month compared to the observations and the other two experiments, which may contributes to the delayed establishment of anomalous WNP-AC (weakened C-mode dynamics), suggesting that the thermal forcing of MC as land surface plays a critical role in the establishment of the anomalous WNP-AC. This result suggests a negative and larger MC thermal forcing on ENSO evolution compared to the MC topographic effect.

It should be noted that the strong ENSO asymmetry in the NOLAND experiment also apparently contributes to the slower decay of its ENSO events, especially the cold events, and hence a lower frequency of ENSO cycle, in addition to the C-mode mechanism we have emphasized. The ENSO asymmetry, especially the long lingering La Niña events, has been examined in previous studies^40–42^. This asymmetry is usually underestimated in climate models (i.e. in the CTL and NOTOPO experiments). Although a detailed analysis on why our NOLAND experiment better depicts this feature is beyond the scope of this paper, it should be an interesting topic for a future study.

In this study, our results emphasize the requirement for a better representation of the topography and hence the convective organization over MC, as mentioned by (ref.^43^). This study also demonstrates the strong physical influence of MC, particularly the MC thermal forcing, on ENSO evolution, which has never been explored previously. It provides a new insight into the understanding, simulation, and prediction of ENSO. Since the anomalous WNP-AC serves as a major mediator to bridge ENSO variability and the climate variation over East Asia^36,44^, the MC impact on anomalous WNP-AC evolution will further contributes to modification of the relationship between ENSO and the East Asia summer monsoon. Therefore, a better MC representation may also lead to improved prediction of the post-ENSO Asian summer monsoon and large-scale seasonal-interannual climate variability.

## Methods

### Reanalysis data sets

The monthly data sets of 925-hPa wind, 10-m wind, and SLP are obtained from the National Centers for Environmental Prediction Climate Forecast System Reanalysis^45^ (NCEP CFSR; https://rda.ucar.edu/datasets/ds093.2) from 1983 to 2005, with a horizontal resolution at 0.5° × 0.5°. The monthly SST is obtained from the National Oceanic and Atmospheric Administration optimally interpolated SST analysis version 2^46^ (NOAA OISST V2; https://www.esrl.noaa.gov/psd/data/gridded/data.noaa.oisst.v2.html) from 1983 to 2005, with a horizontal resolution at 1° × 1°.

### Community Earth System Model (CESM) and experiments

To investigate the MC influences as both dynamical and thermal forcing on ENSO life cycle, we perform three coupled numerical experiments from 1979 to 2005 using the Community Earth System Model (CESM) version 1.2.2^47^. The CESM model consists of four components (atmosphere, ocean, land, and sea ice). The atmospheric model used here is the Community Atmosphere Model version 5 (CAM5) with a finite-volume dynamical core, a nominal 1° horizontal resolution (0.9° × 1.25°), and 30 vertical hybrid levels. The ocean model is the Parallel Ocean Program version 2 (POP2) with a nominal 1° horizontal resolution and 60 vertical levels. The land model is the Community Land Model version 4 (CLM4) with 15 vertical levels. The land and sea ice models share the same horizontal grids with the atmospheric and ocean models, respectively. The experiments use historical, observation-based external forcing data^48^ (e.g. solar irradiance, greenhouse gas concentrations, ozone, and volcanic aerosols). The first experiment uses the U.S. Geological Survey (USGS) 30-second elevation over the globe (referred to as CTL, Supplementary Fig. 1a), while the elevation is reduced to zero over the MC in the second experiment (referred to as NOTOPO, Supplementary Fig. 1b) and the land surface is replaced with ocean surface over the MC with initial conditions extrapolated from the surrounding oceans in the third experiment (referred to as NOLAND). The results from 1983 to 2005 are analyzed.

### Combined empirical orthogonal function analysis

The combined empirical orthogonal functions (CEOF) focus on the coupling between several variables in representing the dominant patterns of temporospatial variability. To assess the representation of dominant atmospheric response related to ENSO variability and the combination mode in the model, a CEOF analysis has been performed on the 925-hPa wind over the tropical Indo-Pacific (90°E–70°W, 30°S–30°N) using reanalysis data, CTL result, NOTOPO result, and NOLAND result, respectively. In other words, the 925-hPa zonal wind and meridional wind are analyzed together in a single empirical orthogonal function analysis, and thus the temporal variations [i.e. principal components (PCs)] by the eigenfunctions are common to both variables. The associated spatial pattern and corresponding time series for each mode, which are orthogonal to those of other modes, yield the amplitude and phase of the variations of monthly-mean 925-hPa wind. Each mode has a unique eigenvalue representing the percentage of variance explained by the specific mode.

### Spectral analysis

We adopt a Tukey window size of 60 months from the Blackman-Tukey (BT) spectral method^49,50^ to calculate the power spectra of the observed and modeled PCs. The spectra are computed by taking the Fourier transform of the lagged autocovariance function, with a maximum of 60 lags and a ratio of taper equal to 0.1. The PC1 and PC2 wind spectra are tested on the 95% red noise confidence level.

### Correlation and significance test

Values of correlation (r) above 0.12, 0.16, and 0.21 are used to estimate the 90%, 95%, and 99% confidence levels for a 23-year length of 1983–2005. Here, *r* is defined as

1 $${\rm{r}}=\frac{{\sum }_{i=1}^{n}({x}_{i}-\bar{x})({y}_{i}-\bar{y})}{\sqrt{{\sum }_{i=1}^{n}{({x}_{i}-\bar{x})}^{2}}\sqrt{{\sum }_{i=1}^{n}{({y}_{i}-\bar{y})}^{2}}},$$

where $\bar{x}$ and $\bar{y}$ are the mean of *x*_*i*_ and *y*_*i*_ from *i* = 1 to *i* = *n* (*n* is the sample size). We compute the statistical significance levels based on the Student’s *t-*test. Here, *t* is defined as

2 $${\rm{t}}=\frac{\bar{x}-\bar{y}}{\sqrt{\frac{(m-1){s}_{1}^{2}+(n-1){s}_{2}^{2}}{m+n-2}}\sqrt{\frac{1}{m}+\frac{1}{n}}},$$

where $\bar{x}$ and $\bar{y}$ are the mean of *x*_*i*_ from *i* = 1 to *i* = *m* and *y*_*i*_ from *i* = 1 to *i* = *n* (m and *n* are the sample sizes), ${s}_{1}^{2}$ and ${s}_{2}^{2}$ are the variances, while *m* + *n* − 2 is the degree of freedom.

### Code availability

The CESM version 1.2.2 code is available via the https://svn-ccsm-models.cgd.ucar.edu/cesm1/release_tags/cesm1_2_2/.

## Electronic supplementary material

Supplementary explanation

## Data Availability

All raw data can be accessed via the links provided above.
