# Supplementary material for: Dynamical and Thermodynamical Influences of the Maritime Continent on ENSO Evolution
Source: Sci Rep. 2018 Oct 18;8:15352. doi: 10.1038/s41598-018-33436-5 (PMC6193933; doi:10.1038/s41598-018-33436-5)
Supplement: Supplementary file 1 — Supplementary explanation [file 41598_2018_33436_MOESM1_ESM.docx]

**Dynamical and Thermodynamical Influences of the Maritime Continent on ENSO Evolution**

Tuantuan Zhang^1^, Bohua Huang^2^*, Song Yang^1, 3, 4^*, Junwen Chen^1^ & Xingwen Jiang^5^

1. School of Atmospheric Sciences, Sun Yat-sen University, Guangzhou, China

2. Department of Atmospheric, Oceanic, and Earth Sciences and Center for Ocean-Land-Atmosphere Studies, George Mason University, Fairfax, Virginia, USA

3. Guangdong Province Key Laboratory for Climate Change and Natural Disaster Studies, Sun Yat-sen University, Guangzhou, China

4. Institute of Earth Climate and Environment System, Sun Yat-sen University, Guangzhou, China

5. Institute of Plateau Meteorology, China Meteorological Administration, Chengdu, China

Submitted to ***Scientific Reports*** in November 2017

Revised September 2018

**Corresponding authors & addresses*: Prof. Song Yang, School of Atmospheric Sciences, Sun Yat-sen University, 135 West Xingang Road, Guangzhou 510275, China, e-mail: [yangsong3@mail.sysu.edu.cn](mailto:yangsong3@mail.sysu.edu.cn); Prof. Bohua Huang, Department of Atmospheric, Oceanic, and Earth Sciences, George Mason University, Fairfax, Virginia 22030, USA, e-mail: bhuang@gmu.edu


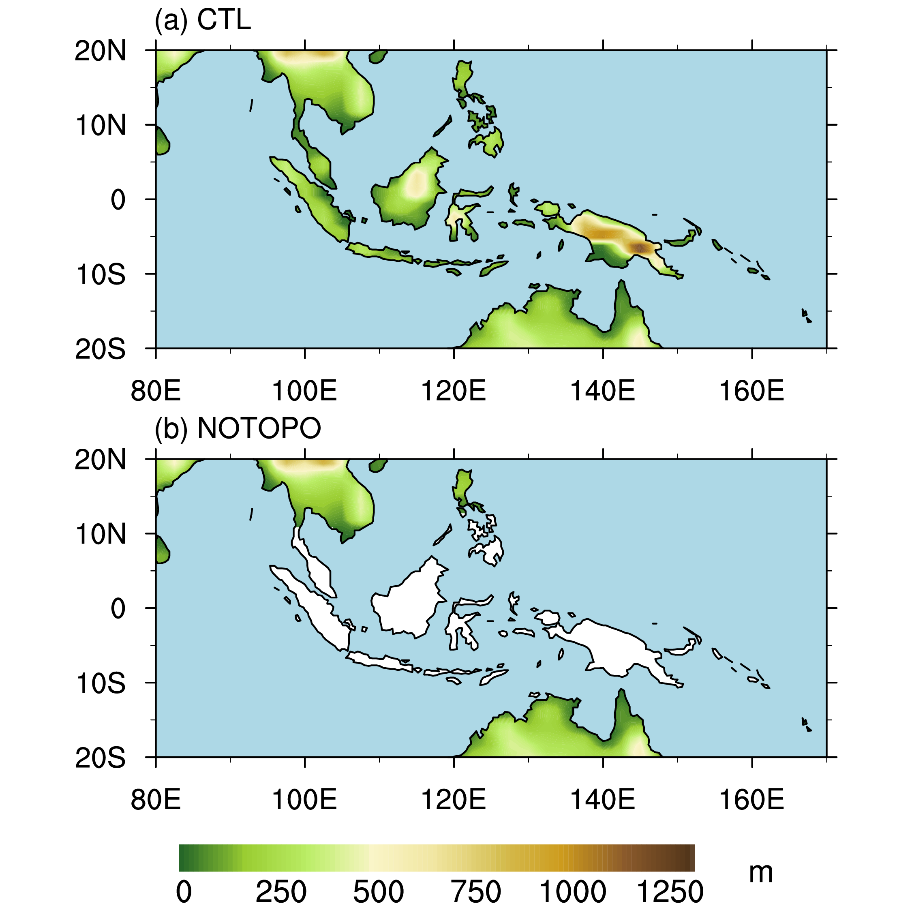


**Supplementary Figure 1 | Topography over the MC.** Elevation of MC in (**a**) CTL experiment and (**b**) NOTOPO experiment. The white shading in (**b**) represents the regions where elevation is reduced to zero in the NOTOPO experiment. In the NOLAND experiment, the topographic elevations are the same as in (**b**) except that the white shaded areas are categorized as ocean surface with initial conditions extrapolated from the surrounding oceans.


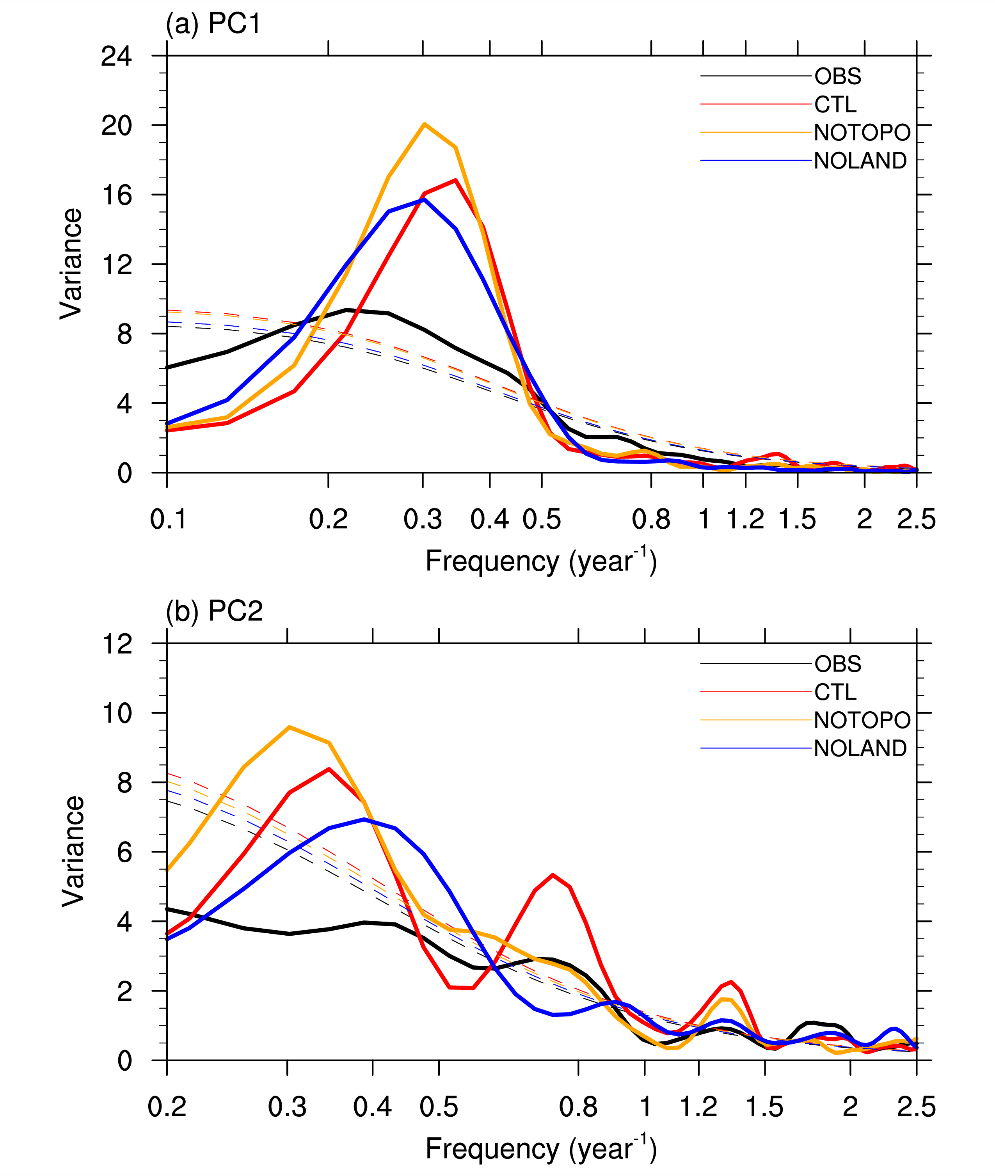


**Supplementary Figure 2 | Changes in frequencies of ENSO and C-mode.** Power spectra for (**a**) first PCs and (**b**) second PCs using the Tukey method. The black, red, orange, and blue solid lines represent PCs for OISST, CTL experiment, NOTOPO experiment, and NOLAND experiment, respectively. The dashed lines denote the corresponding significance at the 95% confidence level.


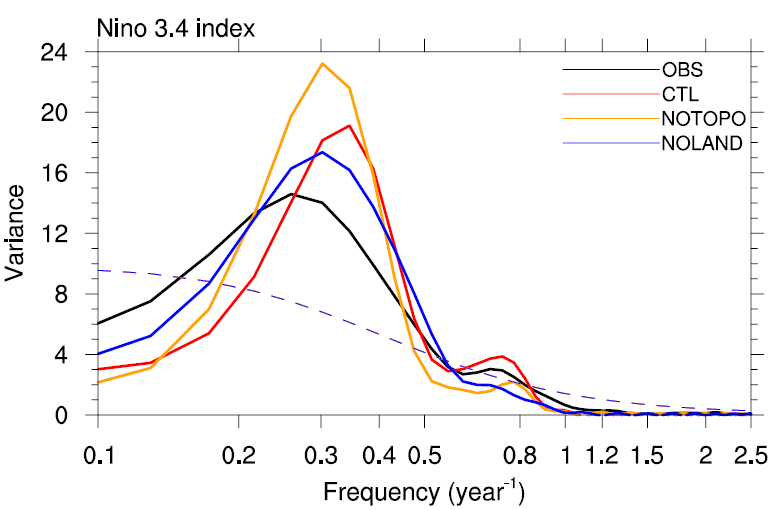


**Supplementary Figure 3 | Changes in ENSO frequency.** The black, red, orange, and blue solid lines represent power spectra for Niño-3.4 indices in OISST, CTL experiment, NOTOPO experiment, and NOLAND experiment, respectively. The dashed lines denote the corresponding significance at the 95% confidence level.


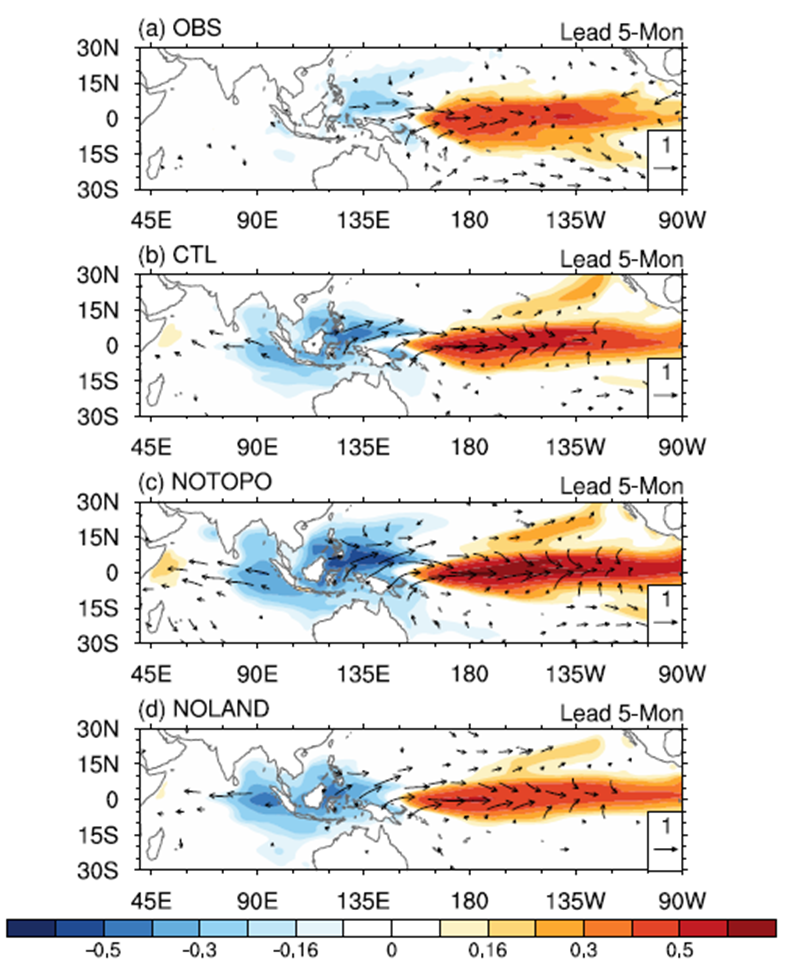


**Supplementary Figure 4 | ENSO devolopment.** Correlations of SST and 10 m wind with the first PCs for lead 5-month from (**a**) observation, (**b**) CTL experiment, (**c**) NOTOPO experiment, and (**d**) NOLAND experiment, respectively. Significant values exceeding the 90% confidence level are shown.


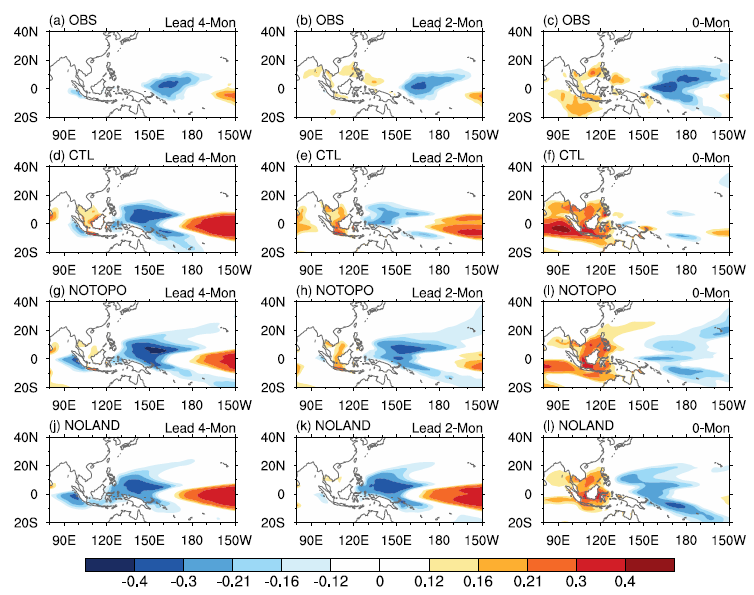


**Supplementary Figure 5 | SST anomalies related to ENSO decaying process.** Correlations of SST with the second PCs for (**left column**) lead 4-month, (**middle column**) lead 2-month, and (**right column**) lag 0-month from (**a-c**) OISST, (**d-f**) CTL experiment, (**g-i**) NOTOPO experiment, and (**j-l**) NOLAND experiment, respectively. Significant values exceeding the 90% confidence level are shown.


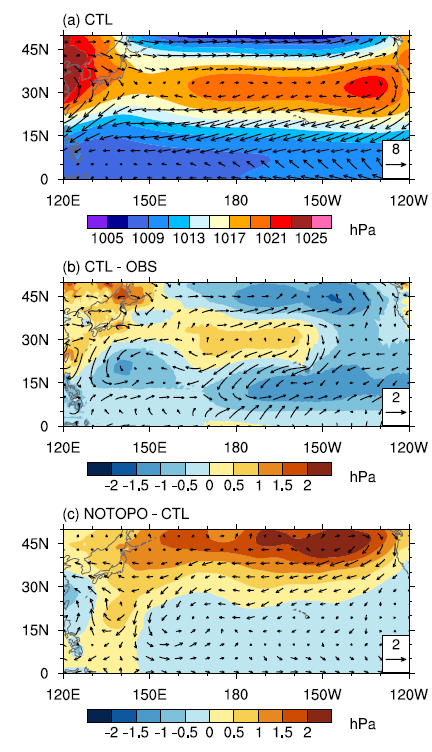


**Supplementary Figure 6 | Improved simulation of seasonal evolution in NOTOPO experiment.** (**a**) Climatological SLP and 10-m wind in November for CTL experiment. (**b**) Differences in SLP and 10-m wind between CTL experiment and CFSR. (**c**) Differences in SLP and 10-m wind between NOTOPO experiment and CTL experiment.
